# *Chlamydia*-Related Bacteria in Free-Living and Captive Great Apes, Gabon

**DOI:** 10.3201/eid2212.150893

**Published:** 2016-12

**Authors:** Anna Klöckner, Michael Nagel, Gilbert Greub, Sébastien Aeby, Karolin Hoffmann, Florian Liégeois, Francois Rouet, Stefania De Benedetti, Nicole Borel, Beate Henrichfreise

**Affiliations:** University of Bonn, Bonn, Germany (A. Klöckner, S. De Benedetti, B. Henrichfreise);; Kumasi Centre for Collaborative Research in Tropical Medicine, Kumasi, Ghana (M. Nagel);; University Hospital Center, Lausanne, Switzerland (G. Greub, S. Aeby);; University of Lausanne, Lausanne (G. Greub, S. Aeby);; University of Zurich, Zurich, Switzerland (K. Hoffmann, N. Borel); Institut de Recherche pour le Développement, Montpellier, France (F. Liégeois);; Université de Montpellier 1, Montpellier (F. Liégeois);; International Centre for Medical Research of Franceville, Franceville, Gabon (F. Liégeois, F. Rouet)

**Keywords:** Chlamydia, bacteria, Chlamydiae, Chlamydiales, great apes, Waddlia chondrophila, Candidatus Rhabdochlamydia, western lowland gorilla, Gorilla gorilla gorilla, central chimpanzee, Pan troglodytes troglodytes, real-time PCR, sequencing, zoonoses, Gabon, central Africa

**To the Editor:** Central Africa is the natural habitat for most of the world’s gorillas and approximately one third of all chimpanzees. As a result of poaching, diseases, and habitat loss, the western lowland gorilla (*Gorilla gorilla gorilla*) and the central chimpanzee (*Pan troglodytes troglodytes*), both referred to as great apes, have been decreasing in numbers since 1970 and are now red-listed by the International Union for Conservation of Nature (*1*). Infectious diseases are major threats to apes in Africa. In addition to Ebola virus disease, a leading cause of death, the health of great apes is compromised by infections with *Bacillus anthracis*, *Staphylococcus aureus*, and *Plasmodium falciparum* (*1*–*4*). Chimpanzees and gorillas are closely related to humans and have similar anatomic, physiologic and immunologic features. Transmission of pathogens from humans to wildlife has been considered a major concern of tourism (*1*).

Except 1 report of bacteria of the order Chlamydiales in a fecal sample from a wild-living Congolese *P. troglodytes* *troglodytes* (*5*), nothing is known about the prevalence of Chlamydiales in great apes. Members of this order are obligate intracellular pathogens that have a unique biphasic life cycle. They infect a wide range of hosts and have major effects on animal and human health worldwide. Until 1993, *Chlamydiaceae* was the only known chlamydial family. However, the discovery of numerous *Chlamydia*-related bacteria species indicated a much broader diversity and host spectrum (*6*). To learn more about the prevalence of Chlamydiales in great apes, we analyzed samples from critically endangered western lowland gorillas and endangered central chimpanzees from Gabon.

We screened 25 samples (8 ocular, 4 vaginal, 7 penile, and 6 rectal swab specimens) obtained noninvasively during routine health checks from 12 apes in captivity. At the time of sampling, the animals were anesthetized and showed no evident signs of disease. All apes were born and reared in captivity at the Primatology Unit of the International Centre for Medical Research of Franceville (Franceville, Gabon) and lived in social groups of ≈10 animals.

We also analyzed feces from wild-living gorillas and chimpanzees, 10 samples from each species, collected in several remote forest areas of Gabon. All samples were collected according to international guidelines used at the International Centre for Medical Research of Franceville. For fecal samples obtained immediately after defecation, the outer layer was removed by using a sterile scalpel, and material from the inner part was frozen to avoid degradation and surface contamination.

Extracted DNA from swab specimens and feces was initially screened for *Chlamydiaceae* by using a 23S rRNA real-time PCR and primers Ch23S-F and Ch23S-R (*7*). A internal control amplification was performed with primers EGFP-1-F and EGFP-10-R, and *Chlamydia abortus* DNA was used to prepare a standard curve.

To detect other Chlamydiales, all samples were analyzed by using a broad-range, pan-Chlamydiales 16S rRNA real-time PCR, which had a sensitivity of 94% and showed no cross-amplification with DNA from other bacterial clades (*8*). Plasmid pCR2.1-TOPO (Invitrogen, Basel, Switzerland), which contained a portion of the 16S rRNA gene targeted by the pan-Chlamydiales 16S rRNA real-time PCR, was used to produce a standard curve. Samples with a cycle threshold <35 were sequenced (GATC Biotech AG, Konstanz, Germany), and results were analyzed by using BLAST (http://blast.ncbi.nlm.nih.gov/Blast.cgi). Purification, real-time PCR, sequencing PCR, and electrophoresis were performed in different laboratories to avoid DNA contamination.

The 16S rRNA real-time PCR and sequencing identified Chlamydiales of the non-*Chlamydiaceae* families in captive and free-living chimpanzees and gorillas. However, we did not identify species in the family *Chlamydiaceae* (Table). For captive great apes, BLAST analysis of 1 rectal (gorilla) and 1 penile (chimpanzee) sample showed 100% and 98% sequence identity, respectively, with *Waddlia chondrophila*. Furthermore, *Candidatus* Rhabdochlamydia sp. cvE88 was found in a vaginal swab specimen of 1 chimpanzee (99% sequence identity) and was still detectable in a second sample from the same site 1 month later. Among free-living apes, 3 of 10 chimpanzee samples were positive for Chlamydiales and showed 96%–99% identity with uncultured Chlamydiales CRG97. One fecal sample from a gorilla contained *W. chondrophila* (100% sequence identity). Chlamydiales detected in urogenital samples might have been acquired through smear infections. For omnivorous chimpanzees, Chlamydiales in fecal samples might have originated from ingestion of infected prey.

**Table:** Analysis of 7 captive and free-living apes for *Chlamydia*-related bacteria by using real-time PCR and sequencing, Gabon*

Ape	Source	Species	Mean C_t_	DNA copies/μL	Closest BLAST† match for 16S rRNA gene	Sequence identity, %	Fragment size, bp	E-value
Cola‡	Rectal swab	*Gorilla gorilla gorilla*	33.02	10.57	*Waddlia chondrophila* WSU 86–1044, complete sequence	100	230	1 × 10^–115^
Cabinda‡	Penile swab	*Pan troglodytes troglodytes*	33.32	8.58	*W. chondrophila* WSU 86–1044, complete sequence	98	241	1 × 10^–111^
Djela‡	Vaginal swab§	*P. troglodytes troglodytes*	29.34	122.41	*Candidatus* Rhabdochlamydia sp. cvE88, partial sequence	99	243	8 × 10^–118^
1882¶	Feces	*P. troglodytes troglodytes*	34.29	8.24	Uncultured Chlamydiales CRG97, partial sequence	98	201	6 × 10^–93^
1883¶	Feces	*P. troglodytes troglodytes *	31.90	43.55	Uncultured Chlamydiales CRG97, partial sequence	99	200	1 × 10^–95^
1885¶	Feces	*P. troglodytes troglodytes*	31.16	73.41	Uncultured Chlamydiales CRG97, partial sequence	96	209	1 × 10^–90^
Gab2130¶	Feces	*G. gorilla gorilla*	35.30	2.38	*W. chondrophila* WSU 86–1044, complete sequence	100	218	5 × 10^–109^

*C_t_, cycle threshold. †http://blast.ncbi.nlm.nih.gov/Blast.cgi ‡Captive ape.  §A second vaginal swab specimen from the same chimpanzee that was collected 1 mo later still showed a positive result by real-time PCR, and sequencing indicated the presence of a *Candidatus* Rhabdochlamydia sp. cvE88. ¶Free-living ape.

We detected members of the order Chlamydiales in great apes from Gabon. Our study not only identified a new chlamydial host but could also help to gain deeper insights into the evolution of Chlamydiales. The emerging pathogen *W. chondrophilia* has been implicated in human and bovine miscarriage and reported to be transmitted zoonotically or after exposure to freshwater amebae infected with *Chlamydia*-related bacteria (*9*,*10*). Further studies are required to determine the prevalence of Chlamydiales in primates and their potential for causing disease in great apes in Africa threatened with extinction.
